# IPC – Isoelectric Point Calculator

**DOI:** 10.1186/s13062-016-0159-9

**Published:** 2016-10-21

**Authors:** Lukasz P. Kozlowski

**Affiliations:** Kielce, 25-430 Poland

**Keywords:** Isoelectric point, Proteomics, *pKa* dissociation constant

## Abstract

**Background:**

Accurate estimation of the isoelectric point (*pI*) based on the amino acid sequence is useful for many analytical biochemistry and proteomics techniques such as 2-D polyacrylamide gel electrophoresis, or capillary isoelectric focusing used in combination with high-throughput mass spectrometry. Additionally, *pI* estimation can be helpful during protein crystallization trials.

**Results:**

Here, I present the Isoelectric Point Calculator (IPC), a web service and a standalone program for the accurate estimation of protein and peptide *pI* using different sets of dissociation constant (*pKa)* values, including two new computationally optimized *pKa* sets. According to the presented benchmarks, the newly developed IPC *pKa* sets outperform previous algorithms by at least 14.9 % for proteins and 0.9 % for peptides (on average, 22.1 % and 59.6 %, respectively), which corresponds to an average error of the *pI* estimation equal to 0.87 and 0.25 pH units for proteins and peptides, respectively. Moreover, the prediction of *pI* using the IPC *pKa*’s leads to fewer outliers, i.e., predictions affected by errors greater than a given threshold.

**Conclusions:**

The IPC service is freely available at http://isoelectric.ovh.org Peptide and protein datasets used in the study and the precalculated *pI* for the PDB and some of the most frequently used proteomes are available for large-scale analysis and future development.

**Reviewers:**

This article was reviewed by Frank Eisenhaber and Zoltán Gáspári

**Electronic supplementary material:**

The online version of this article (doi:10.1186/s13062-016-0159-9) contains supplementary material, which is available to authorized users.

## Background

Analysis of proteins starts from the heterogeneous mixture (lysate) from which protein fraction needs to be isolated. Next, individual proteins are separated and finally identified. The procedure relies on physicochemical properties of amino acids such as a molecular mass or a charge. Over the years, many techniques were introduced to allow to accomplish the task. One of the oldest, but still widely used technique is 2-D polyacrylamide gel electrophoresis (2D-PAGE) [1, 2], where proteins are separated in two dimensions on a gel and identified using estimated molecular weight and isoelectric point (*pI* is the pH value at which the net charge of a macromolecule is zero, and therefore its electrophoretic mobility is stopped). Unfortunately, 2D-PAGE suffers from several intrinsic technical problems (e.g., performs poorly for very large, very small, extremely acidic or basic proteins). Therefore, 2D-PAGE has been today replaced in many cases by gel-free techniques such as high-throughput mass spectrometry (MS) [3, 4]. Nevertheless, before the mass spectrometry is applied, the sample is digested by trypsin into short peptides and then fractionated by isoelectric focusing into so called fractions which allows to reduce MS analysis complexity. Although molecular techniques for protein analysis have changed, the interpretation of the results from those techniques in many cases rely on accurate estimations of *pI* for reference polypeptides.

For polypeptides, *pI* depends mostly on the acid dissociation constants (*pKa)* of the ionizable groups of seven charged amino acids: glutamate (δ-carboxyl group), aspartate (ß-carboxyl group), cysteine (thiol group), tyrosine (phenol group), histidine (imidazole side chains), lysine (ε-ammonium group) and arginine (guanidinium group). Additionally, the charge of the amine and carboxyl terminal groups contribute to *pI* and can greatly affect *pI* of short peptides [5]. Overall, the net charge of the protein or peptide is strongly related to the solution (buffer) pH and can be approximated using the Henderson-Hasselbalch equation [6]. It should be kept in mind that the values of dissociation constants used in the calculations are usually derived empirically and can vary substantially depending on the experimental setup such as temperature or buffer ionic strength (herein presented method, Isoelectric Point Calculator, is compared to 15 such *pKa* sets). On the other hand, *pKa* values or *pI* can be derived computationally giving the large sets of proteins or peptides for which *pI* information is known. This is the approach, presented in this study. The problem of computational prediction of *pI* was already addressed by two other research groups using artificial neural networks (ANN) [7] and support vector machines (SVM) [8, 9]. Here, I present IPC program which is based on the optimization using a basin-hopping procedure [10]. Presented results shows that IPC overperform all currently, available algorithms.

## Results

### Comparison to other algorithms

To compare the performance of Isoelectric Point Calculator 15, other *pKa* sets and two programs based on SVM (pIR) and ANN (pIPredict) were tested. Isoelectric point predictions were validated separately for peptides and proteins as they differ substantially. Proteins are relatively big molecules with a plethora of charged residues. Moreover, in the proteins *pI* is affected by many, additional factors such as post translational modifications, solvent accessibility, etc. On the other hand, peptides are short, possessing usually only a handful of charged residues and therefore their *pI* is easier to predict. In the presented study two protein databases, SWISS-2DPAGE and PIP-DB, were used. For peptides, three datasets from separate high-throughput experiments were used. At the beginning, two databases for proteins were merged. As the content of the databases overlapped and was redundant, additional post processing and cleaning of the data was necessary. First of all, not all records contained useful information, namely isoelectric point and sequence or Uniprot ID. Moreover, even separate databases were redundant (contained multiple records with the same sequence or Uniprot ID). Therefore, the duplicates were merged into unique records and *pI* information was averaged if needed (multiple *pI* values coming from separate experiments). Next, the worst outliers defined here as those proteins for which the difference between the experimental *pI* and the average predicted *pI* was greater than the threshold of the mean standard error (MSE) of three were excluded as they represented possible annotation errors. Finally, the resulting dataset consisting of over 2,000 proteins was divided into a training set (75 % randomly chosen proteins) and a testing set. The training set was used to obtain optimized *pKa* values and the test set was used to evaluate IPC on proteins not used during training. A similar procedure was employed to peptide datasets with the exception that then the threshold of MSE of 0.25 was used (for more details see Methods). The results of the benchmarks for *pI* prediction are presented in Tables 1, 2 and 3. Table 1 shows the results on testing sets both for proteins and peptides. IPC produced best results (the lowest RMSD and the smallest number of outliers). For comparison the results on the training set are presented in Table 2. The performance of the IPC_protein set is slightly better for the training dataset (RMSD of 0.8376 for the 75 % training set versus 0.8731 for the 25 % test set), but this is expected (even though optimization procedure was cross validated the overfitting cannot be avoided fully, but results in Tables 1 and 2 show that this is not critical in this case). Moreover, the general performance of IPC does not depend on the datasets used for training (Table 3). Furthermore, the results for the training sets and the results for the test sets are consistent (Tables 1 and 2, respectively). In most cases the order of the method’s performance on both training and testing datasets is similar; for instance the change in the order on the protein dataset can be seen for the Dawson and Bjellqvist *pKa* sets, which is within the error margin. Similarly, there are some changes in the method order depending on the peptide dataset, but only for methods with a very similar performance, e.g., Lehninger and Solomons on PIP-DB. Again, in most cases, the change is within the margin of error. The IPC sets, regardless of the dataset and the validation procedures, performed the best. Similar results are obtained when comparing the number of outliers produced by the individual *pKa* sets. Outliers correspond to cases of extremely poor prediction (the difference between the predicted and experimental pI is greater than an arbitrarily chosen threshold; e.g., for proteins, an MSE of 3 was used as the threshold). In all cases, IPC produced the smallest number of outliers. It should be stressed, that all algorithms, except IPC, pIR and pIPredict, rely on experimentally derived *pKa* values and therefore they were not optimized for particular data sets. As IPC results were validated on test set not used in training, the only remaining algorithms which may be optimized towards a particular dataset are pIR and pIPredict. pIR is a support vector machine method which used PIP-DB proteins for training, thus it is interesting to investigate how it performs on different protein set. As one can see in Table 3, while pIR produce reasonable results for the PIP-DB dataset, its predictive performance decreases significantly on the SWISS-2DPAGE dataset. This means that pIR method was most likely overfitted towards PIP-DB proteins (move from the middle of the table – PIP-DB dataset, to the bottom – SWISS-2DPAGE dataset). Moreover, it should be stressed that all benchmarks from Audain et al. and presented here describing PIP-DB cannot be compared directly as they were done on different subsets of PIP-DB (Audain et al. removed all records which have more than one *pI* measurement for given protein, while here average was used instead). Also, pIPredict performs worse than most of the methods. Most likely it is due the fact that pIPredict was trained only on peptide dataset from Gauci et al., which is smaller than used in the presented study. Moreover, it was not trained on any protein dataset, thus pIPredict should rather be used only for peptides.

**Table 1:** Prediction of isoelectric point on the 25 % testing datasets

Method	Protein dataset			Method	Peptide dataset		
	RMSD	%	Outliers		RMSD	%	Outliers
IPC_protein	0.874	0	46	IPC_peptide	0.251	0	232
Toseland	0.934	14.9	52	Solomons	0.255	0.9	235
Bjellqvist	0.944	17.7	47	Lehninger	0.262	2.5	236
Dawson	0.945	17.8	56	EMBOSS	0.325	18.5	372
Wikipedia	0.955	20.5	55	Wikipedia	0.421	47.9	1467
Rodwell	0.963	22.8	58	Toseland	0.425	49.1	990
ProMoST	0.966	23.6	52	Sillero	0.428	50.3	1223
Grimsley	0.968	24.2	60	Dawson	0.435	52.9	1432
Solomons	0.970	24.8	58	Thurlkill	0.481	69.7	1361
Lehninger	0.970	25.0	59	Rodwell	0.502	78.4	1359
pIR	1.013	38.0	58	DTASelect	0.550	99.1	1714
Nozaki	1.024	41.3	56	Nozaki	0.602	124.3	1368
Thurlkill	1.030	43.4	61	Grimsley	0.616	131.4	1550
DTASelect	1.032	44.1	58	Bjellqvist	0.669	161.5	1583
pIPredict	1.048	49.4	56	pIPredict	1.024	493.6	2720
EMBOSS	1.056	52.3	69	ProMoST	1.239	873.4	2649
Sillero	1.059	53.2	63	pIR	1.881	4159.7	3358
Patrickios	2.392	3201.8	227	Patrickios	1.998	5479.1	2739
Avg_pI^a^	0.960	22.1	53	Avg_pI	0.454	59.6	1571

^a^Average from all *pKa* sets without Patrickios (highly simplified *pKa* set) and IPC sets. Note, that the average *pI* is calculated on the level of individual protein or peptide, thus it does not represent the average from values presented in the table for individual methods

% - Note that the pH scale is logarithmic with base 10; thus, the percent difference corresponds to pow(10, x), where x is equal to the delta of the RMSD of two error estimates represented in pH units; for example, the % difference between Toseland and IPC_protein is pow(10, (0.934-0.874))

Protein dataset (IPC_protein was trained on 1,743 proteins with 10-fold cross-validation – data in Table 2, tested on 581 proteins not used for training – data in the table above), peptide dataset (IPC trained on 12,662 peptides with 10-fold cross-validation – data in Table 2, tested on 4,220 peptides not used for training – data in the table above). Outliers correspond to the number of predictions for which the difference between the experimental *pI* and predicted pI was greater than the threshold of the mean standard error (MSE) of 3 for the protein dataset and MSE of 0.25 for the peptide dataset

**Table 2:** Prediction of isoelectric point on the 75 % training datasets

Method	Protein dataset			Method	Peptide dataset		
	RMSD	%	Outliers		RMSD	%	Outliers
IPC_protein	0.838	0	114	IPC_peptide	0.247	0	635
Toseland	0.898	15.0	131	Solomons	0.251	0.8	638
**Bjellqvist**	**0.922**	**21.5**	**149**	Lehninger	0.256	2.4	643
**Dawson**	**0.920**	**20.9**	**156**	EMBOSS	0.322	18.8	1088
Wikipedia	0.930	23.8	157	Wikipedia	0.413	46.3	4280
Rodwell	0.938	26.1	159	Sillero	0.426	50.9	3025
ProMoST	0.938	26.1	140	Toseland	0.427	51.2	3618
Grimsley	0.939	26.2	147	Dawson	0.432	52.9	4192
**Solomons**	**0.947**	**28.5**	**159**	Thurlkill	0.480	70.8	4017
**Lehninger**	**0.947**	**28.7**	**160**	Rodwell	0.506	81.2	4061
**pIR**	**1.026**	**54.2**	**180**	DTASelect	0.541	96.8	4902
**Nozaki**	**1.005**	**47.1**	**169**	Nozaki	0.599	124.8	4013
**Thurlkill**	**1.018**	**51.5**	**173**	Grimsley	0.611	130.9	4609
**DTASelect**	**1.017**	**51.1**	**167**	Bjellqvist	0.661	159.2	4672
**pIPredict**	**1.057**	**65.9**	**173**	pIPredict	1.024	497.8	8051
EMBOSS	1.040	59.4	189	ProMOST	1.233	867.5	7999
Sillero	1.042	60.1	188	pIR	1.862	4020.9	9921
Patrickios	2.237	2405.1	645	Patrickios	1.977	5266.8	8131
Avg_pI^a^	0.940	26.6	151	Avg_pI	0.451	59.7	4600

^a^Average from all *pKa* sets without the Patrickios (highly simplified *pKa* set) and IPC sets. Note, that the average *pI* is calculated on the level of individual protein or peptide

Protein dataset (IPC_protein trained on 1,743 proteins with 10-fold cross-validation – data in the table above, tested on 581 proteins not used for training – data in Table 1), peptide dataset (IPC trained on 12,662 peptides with 10-fold cross-validation – data in above table, tested on 4,220 peptides not used for training – data in Table 1). Changes in method order in comparison to Table 1 are in bold

Outliers correspond to the number of predictions for which the difference between the experimental *pI* and the predicted *pI* exceeded the threshold of an MSE of 3 for the protein dataset and an MSE of 0.25 for the peptide dataset

**Table 3:** Prediction of isoelectric points for SWISS-2DPAGE and PIP-DB databases

Method	SWISS-2DPAGE			Method	PIP-DB		
	RMSD	%	Outliers		RMSD	%	Outliers
IPC_protein	0.476	0	10	IPC_protein	1.019	0	141
Toseland	0.521	10.9	18	Toseland	1.086	16.7	153
Bjellqvist	0.590	30.0	31	Bjellqvist	1.085	16.3	150
ProMoST	0.597	32.1	29	Dawson	1.081	15.3	161
Dawson	0.599	32.5	37	Wikipedia	1.087	16.9	163
Wikipedia	0.619	39.0	35	Rodwell	1.095	19.1	167
Rodwell	0.628	41.7	37	Grimsley	1.121	26.6	170
Grimsley	0.572	24.5	21	Solomons	1.103	21.4	159
Solomons	0.635	44.2	44	Lehninger	1.102	21.1	161
Lehninger	0.640	45.8	44	ProMOST	1.111	23.5	150
Nozaki	0.679	59.4	43	pIR	1.152	35.8	184
Thurlkill	0.691	63.9	39	Nozaki	1.165	39.9	170
DTASelect	0.677	58.8	35	Thurlkill	1.180	44.9	176
EMBOSS	0.724	76.9	49	DTASelect	1.186	47.1	173
Sillero	0.721	75.5	50	pIPredict	1.195	50.0	182
pIR	0.761	92.4	37	EMBOSS	1.198	51.2	191
pIPredict	0.768	95.9	33	Sillero	1.202	52.4	187
Patrickios	1.600	1227.9	243	Patrickios	2.623	3918	604
Avg_pI^a^	0.614	37.1	32	Avg_pI^a^	1.101	20.9	160

^a^Average from all *pKa* sets without the Patrickios (highly simplified *pKa* set) and IPC sets. Note, that the average *pI* is calculated on the level of individual protein or peptide

Both SWISS-2DPAGE and PIP-DB were cleaned of outliers (MSE > 3 between experimental *pI* and average predicted *pI*) and clustered by CD-HIT with 99 % sequence identity threshold, as described in the Materials and Methods (982 and 1,307 proteins, respectively), but they were not divided into training and testing datasets. Thus, the results for the IPC sets are slightly overestimated, but this is not relevant, as shown by the comparison of Tables 1 and 2

Outliers correspond to the number of predictions for which the difference between the experimental *pI* and the predicted *pI* exceeded the threshold of an MSE of 3 for the protein dataset

### Auxiliary statistics

**Fig. 1:**
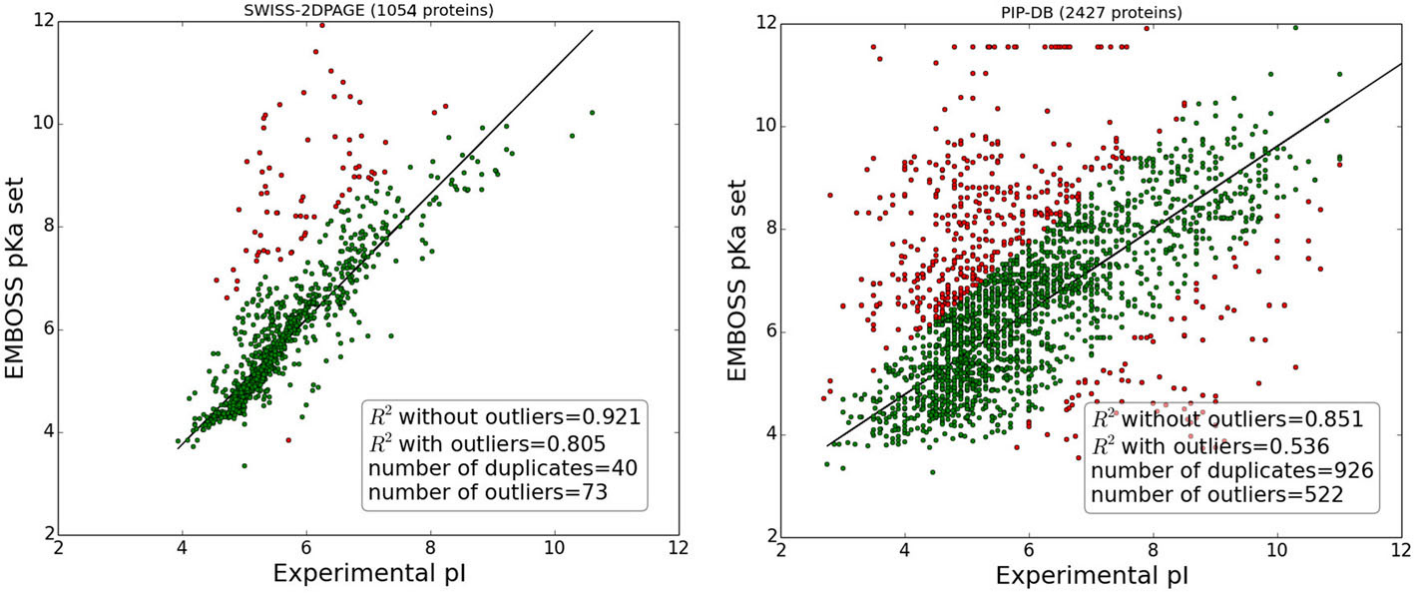
Correlation of the experimental versus the theoretical isoelectric points for two protein datasets. Data for SWISS-2DPAGE (*left panel*) and PIP-DB (*right panel*) are calculated using the EMBOSS *pKa* set. Outliers are defined as MSE > 3 and are marked in red. Plots correspond to datasets as presented by the authors before cleaning and the removal of duplicates (duplicates are defined as records that have the same sequence but are referred to as separate records in the database). In both databases, the authors reported multiple *pI* values from different experiments for the same sequences in separate records. In such cases for the current analysis, the average *pI* was used. The solid line represents the linear regression after removal of the outliers

**Fig. 2:**
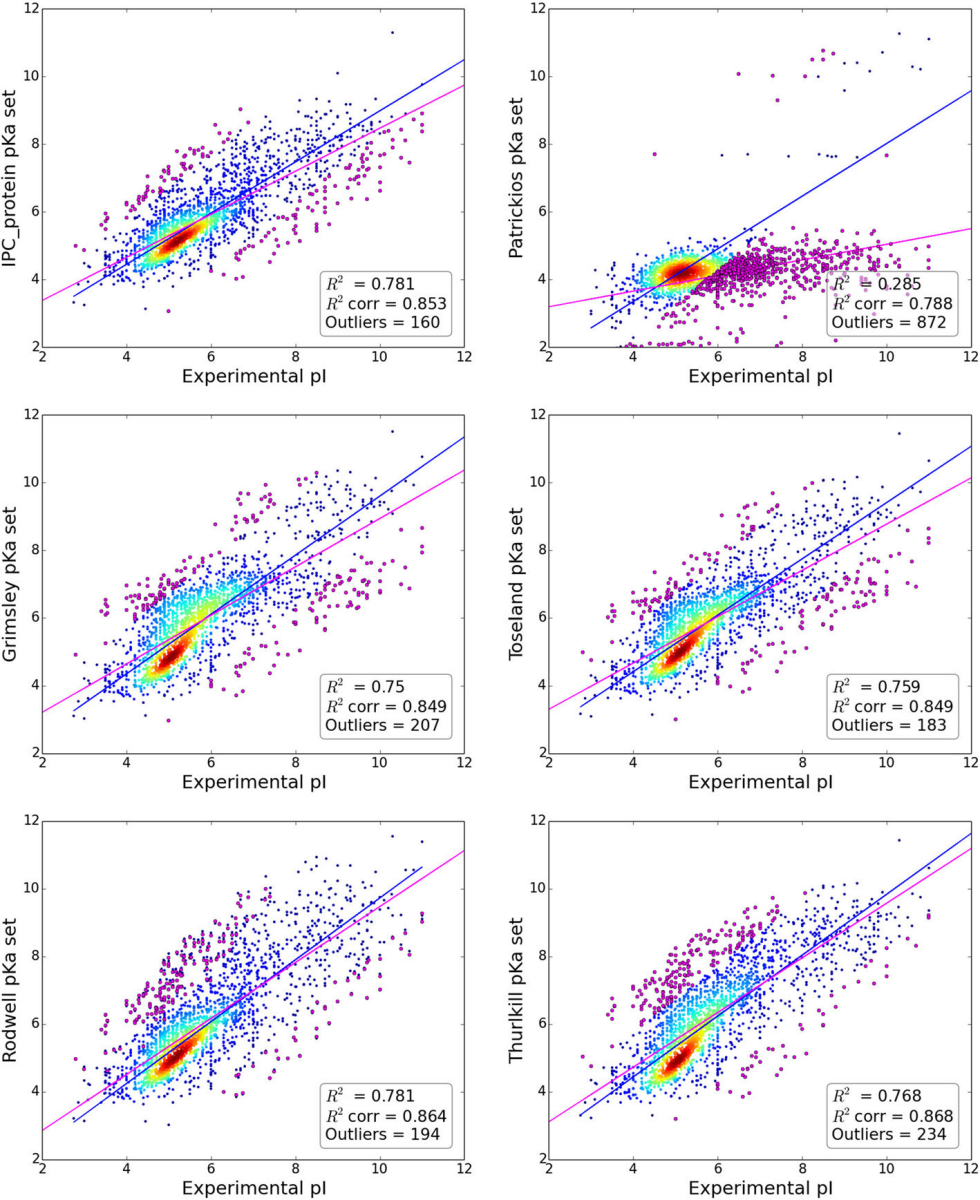
Correlation of the experimental versus theoretical isoelectric points calculated using different *pKa* sets. Data for the main protein dataset (merged dataset created from SWISS-2DPAGE and PIP-DB). R^2^ – Pearson correlation before the removal of outliers. R^2^corr – Pearson correlation after the removal of outliers. Additionally, the linear regression models fitted to predictions with outliers (*magenta line*) and without outliers (*blue line*) are shown. Outliers (*marked in magenta*) are defined as *pI* predictions with MSE > 3 in comparison to the experimental *pI*. Other predictions are represented as heat maps according to the density of points. The numbers of outliers for both the training and testing set are shown together. For brevity, only six *pKa* sets are shown

Figures 1 and 2 show the correlation plots between the experimental and theoretical isoelectric points for proteins and peptides on different datasets calculated using different *pKa* sets. These plots are useful to assess the quality of the datasets used. The Pearson correlations (R^2^) between a *pKa* set, e.g., EMBOSS and the number of outliers, which were defined here as those where the MSE exceeded 3 for the average pI prediction (this corresponds to ~1.73 pH unit difference) give a good impression of the quality of the dataset. Even if we assume that the presented, nine-parameter model is highly simplified e.g., it does not take posttranslational modifications into account, we can suspect that such a large difference is more likely an annotation error in the database than a true difference (this assumption was confirmed by randomly checking some outliers; data not shown, available on request). Moreover, contrary to previous works, R^2^ was not used as a performance measure because it should not be considered in this way. R^2^ measures how well the current model fits a linear model. It is unlikely that the experimental isoelectric point can be explained using a highly simplified nine-parameter model that does not take into account multiple factors (see Methods for more details). The R^2^ value is a useful statistic for preliminary analysis but should not be used for evaluating the performance. Similarly, scatter plots between the experimental *pI* and those produced by different *pKa* sets (Fig. 2) can give a good impression of the correctness of the model, but quantitative measurement of the performance requires better measures, e.g., the root-mean-square deviation (RMSD), which presents the sample standard deviation of the differences between the predicted values and the observed values. An additional advantage of the RMSD is that it is simple to explain and reflects the error of the prediction in pH units. Another performance metric used here is the number of outliers at a given threshold (for the protein dataset the threshold was set to MSE > 3 between the experimental *pI* and average prediction *pI* for removing outliers from the datasets; in this way, none of the *pKa* sets was favored). For instance, the Patrickios *pKa* set is highly simplified and generally should not be used. Thus, this set was not included in the average calculation. In all benchmarks, the Patrickios *pKa* set performed the worst. As illustrated in Fig. 2 (top, right panel), this set cannot correctly predict the *pI* for proteins with *pI* > 6, but it performs relatively well in the 4–6 *pI* range.

## Discussion

**Fig. 3:**
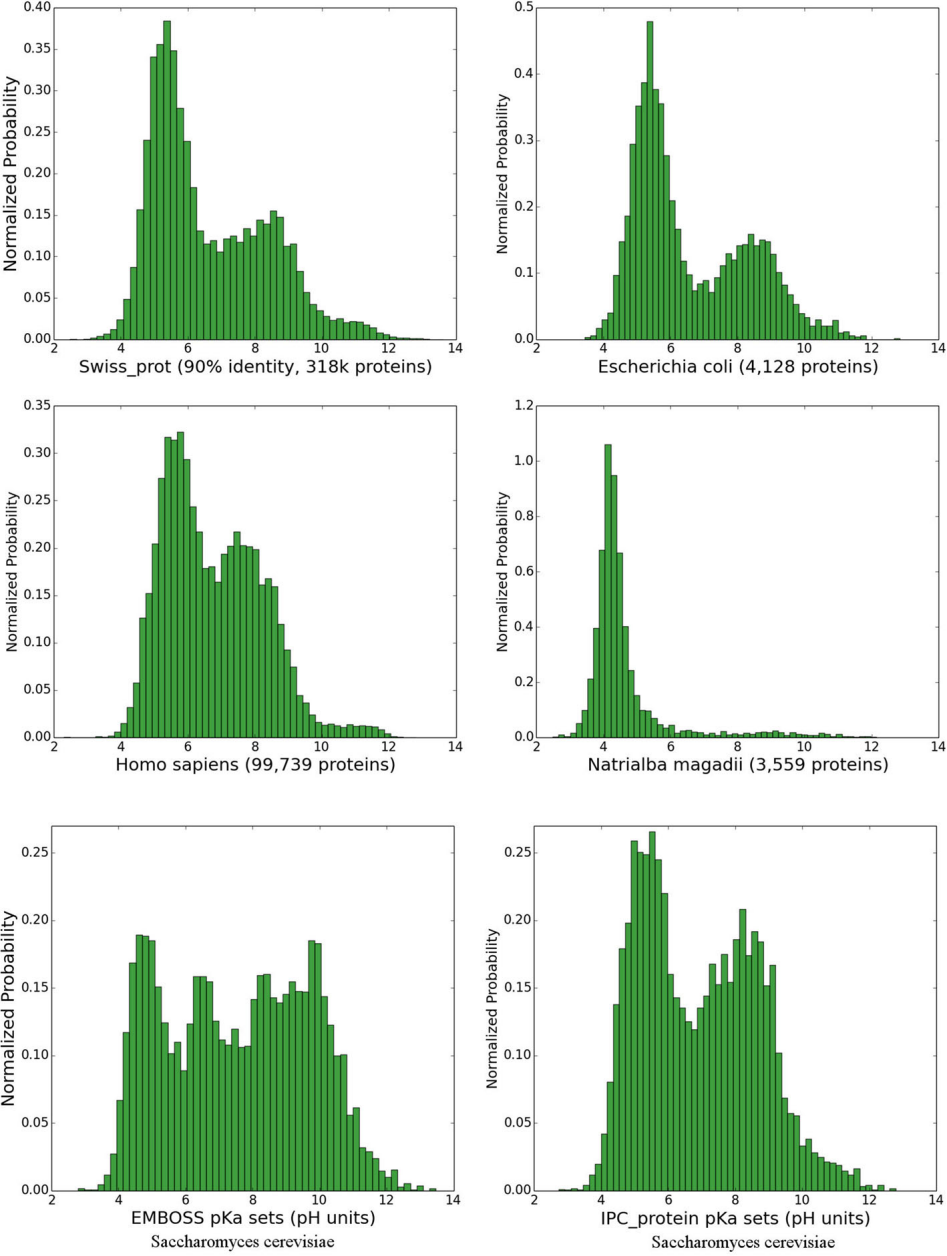
Histograms of the isoelectric points of proteins. *Top* and *middle* panels are calculated using the IPC_protein *pKa* set (in 0.25 pH unit intervals) and represents *pI* distribution in the *SwissProt* database, human proteome, *Escherichia coli* and extreme halophilic archaeon *Natrialba magadii*. Bottom two panels presents the isoelectric points of the yeast proteome (6,721 proteins) calculated using the EMBOSS *pKa* set (as presented in the Saccharomyces Genome Database [40]) and the IPC_protein *pKa* set for comparison

The distribution of the isoelectric points of proteins in proteomes is universal for almost all organisms [11], which can be demonstrated by plotting isoelectric points of the proteins stored in the *SwissProt* database. The distribution is bimodal with a low fraction of proteins with a *pI* close to 7.4. This is because the proteins are mostly insoluble, less reactive and unstable at pH close to their *pI*. The pH inside of most cells is close to 7.4, therefore this property of proteomes can be a result of evolutionary selection or simply a result of the chemical properties of amino acids [12]. Naturally, there are some exceptions. Some halophilic Archaea organisms do not try to fight the high concentration of salt in their environment; instead, they change the physiological pH inside their cells to be more similar to the environment (in this way, they use less energy to maintain homeostasis) [13]. This response has dramatic consequences for the amino acid compositions and isoelectric points of their proteins (Fig. 3).

It should be stressed that the relative difference between the performance of different *pKa* sets is often small and statistically insignificant (e.g., *pI* calculated by Bjellqvist vs. Dawson *pKa* sets on protein datasets), but even general knowledge of which *pKa* sets are better and which should be used for a particular type of data (e.g., protein versus peptides) is not commonly used (Fig. 3, bottom two panels). Furthermore, presented results demonstrate that prediction of *pI* is easier for short peptides than for proteins as the former contain less charged and modified amino acids (e.g. compare RMSD values between peptide and protein datasets). Similarly, the dataset on which methods are trained and/or evaluated can result in different estimations of RMSD error. For example, Fig. 1 shows that PIP-DB contains multiple outliers and duplicates in comparison to SWISS-2DPAGE. This noise in the data leads to almost a doubling of the RMSD (Table 3). Nevertheless, the method order is usually preserved.

As mentioned earlier, one of the main limitations of IPC is that it uses a nine-parameter model which is a highly simplistic approximation, and does not take into account many aspects of proteins such as post translational modification. It should be noted that posttranslational modifications occur much more frequently in Eukaryotic proteins than in Prokaryotic, thus it is interesting to investigate how accurately *pI* can be predicted in these two kingdoms separately. As illustrated in Additional file 1: Table S1 all *pI* prediction methods perform better on prokaryotic proteins. This suggests that when working with Eukaryotic proteins one should keep in mind that *pI* prediction accuracy can be decreased due possible posttranslational modifications. In such cases other, more specialized programs such as ProMoST can be used when researcher has detailed knowledge about posttranslational modifications.

Additional source of bias may come from the fact that some proteins can have more than one splicing variant, while in herein study only first, major isoform of protein was used. Thus, there is possibility that this may lead to dataset of proteins different than those for which *pI* was measured. As illustrated in Additional file 1: Table S2 most of analyzed proteins possess only one isoform (2,106 out of 2,254 in the protein dataset, 93.6 % of cases) and when reanalyzing the data using only those proteins the results are virtually identical. It should be stressed that even for proteins having more than one splicing isoform it is highly unlikely that the authors worked with and then reported *pI* from less abundant, minor isoforms.

Moreover, if is easy to notice that presented here new *pKa *values are different from those which were derived earlier experimentally. One should remember that even experimental setup can have strong impact on the results. For instance *pKa *values obtained by Thurkill et al. were measured using alanine pentapeptides with charged residue in the center. This was done to minimalize the contribution from neighboring residues, but this setup is extremely far from the real situation in the proteins (contribution from surrounding side groups of residues which are not alanine, post translational modifications, etc.). Thus, optimized *pKa*’s can be seen as more precise as they indirectly take into account such complexity. In the Additional file 1: Table S3 one can find average *pKa* values from previously used scales compared to IPC values. On peptide dataset most of differences is due terminal residues, which could be expected as in the peptides terminal charge can constitute big proportion of overall charge, thus N-terminus *pKa* value in previous studies was underestimated, while C-terminus *pKa* was overestimated in comparison to IPC values. On the other hand, for proteins one can notice that the main differences are observed for cysteines reflecting possible contribution from disulfide bridges and for lysine, histidine, and tyrosine which are frequently posttranslationally modified. Moreover, this effect is less abundant for arginine (also frequently modified), but it should be noted that arginine is bigger and contains more charged groups thus most likely modification effect (if exists) is less profound.

## Conclusions

New, herein presented *pKa* sets, optimized computationally, can be considered as important improvement in isoelectric point estimation based only on sequence information. IPC had been compared to numerous methods, including 15 other *pKa* sets, two machine learning approaches and the consensus. Datasets used in the study were crossvalidated during training and additionally performance was measured on 25 % subsets not used during training. In all cases, IPC produced superior results. For instance, the isoelectric point prediction algorithm performance measured on proteins derived from different databases (Table 3) differ in absolute value (measurements done on different proteins), but the overall order of methods in the benchmark stays almost the same with IPC leading in all cases. The same is true if we divide datasets according to organism (for details see Additional file 1: Figure S1) from which proteins come. As expected, for all methods the prediction accuracy is decreased for Eukaryotic proteins as they can be frequently posttranslationally modified in contrast to Prokaryotic proteins in which posttranslational modifications are less abundant (Additional file 1: Table S1). As there is no information about posttranslational modifications in used databases (SWISS-2DPAGE and PIP-DB) it was not possible to investigate this issue in more detail. Yet, both separation of proteins into Eukaryotic vs. Prokaryotic and detailed analysis of new *pKa* values shows that the potential bias coming from posttranslationally modification was partially incorporated during optimization procedure which changed *pKa* values mostly for amino acids frequently modified.

To Authors’ knowledge IPC web server is the only website on which protein isoelectric point can be predicted using so many different *pKa* values sets including two, new ones presented here. Accurate estimation of isoelectric point is frequently used for identification of proteins during 2D-PAGE and mass spectrometry. Moreover, the knowledge of isoelectric point can be useful during crystallization trials [14].

## Methods

### Isoelectric point, Henderson–Hasselbalch equation, *pKa* values for the ionizable groups of proteins

The isoelectric point (*pI*) is the pH at which the net charge of a protein is zero. For polypeptides, the isoelectric point depends primarily on the dissociation constants (*pKa)* for the ionizable groups of seven charged amino acids: glutamate (δ-carboxyl group), aspartate (ß-carboxyl group), cysteine (thiol group), tyrosine (phenol group), histidine (imidazole side chains), lysine (ε-ammonium group) and arginine (guanidinium group). Moreover, the charge of the terminal groups (NH_2_ and COOH) can greatly affect the *pI* of short peptides. Generally, the Glu, Asp, Cys, and Tyr ionizable groups are uncharged below their *pKa* and negatively charged above their *pKa*. Similarly, the His, Lys, and Arg ionizable groups are positively charged below their *pKa* and uncharged above their *pKa* [5]. This has certain implications. For example, during electrophoresis, the direction of protein migration on the gel depends on the charge. If the buffer pH (and as a result, the gel pH) is higher than the protein isoelectric point, the particles will migrate to the anode (negative electrode), and if the buffer pH is lower than the isoelectric point, they will migrate to the cathode. When the gel pH and the protein isoelectric point are equal, the proteins stop to migrate.

Overall, the net charge of the protein or peptide is related to the solution (buffer) pH. We can use the Henderson-Hasselbalch equation [6] to calculate the charge at a certain pH:

- for negatively charged residues:

1 $$ {\displaystyle \sum_{i=1}^n\frac{-1}{1+{10}^{pKn-pH}}} $$

where *pKn* is the acid dissociation constant of the negatively charged amino acid

- for positively charged residues:

2 $$ {\displaystyle \sum_{i=1}^n\frac{1}{1+{10}^{pH-pKp}}} $$

where *pKp* is the acid dissociation constant of the positively charged amino acid

The charge of a macromolecule at a given pH is the sum of the positive and negative charges of the individual amino acids given by Eqs. 1 and 2. When the *pKa* values are set, the only variable in the equations is the pH of the buffer, and by iteratively changing the pH, we can easily calculate the isoelectric point. The result will be almost certainly different than the real isoelectric point because many proteins are chemically modified (e.g., amino acids can be phosphorylated, methylated, acetylated), which can change their charge. The occurrence of cysteines (negative charge), which may oxidize and lose charge when they form disulfide bonds in the protein, is also problematic. Moreover, one must consider the charged residue exposure to solvent, dehydration (Born effect), charge-dipole interactions (hydrogen bonds), and charge-charge interactions [5].

Nevertheless, the most critical consideration for accurate isoelectric point determination is the use of appropriate *pKa* values. Unfortunately, *pKa* estimates differ depending on the experimental setup in which they were measured. More than 600 different *pKa* values have been reported for the ionizable groups [15]. Table 4 shows the most commonly used values, including two new *pKa* sets (IPC_protein and IPC_peptide) proposed in this study. Most of the algorithms use nine-parameter model (seven *pKa* values corresponding to charged amino acids and two for the terminal groups), but more advanced algorithms also exist, e.g., Bjellqvist [16] (17 parameters) and ProMoST [17] (72 parameters), which take advantage of specifying additional *pKa* values for charges of particular amino acids, especially those located on the polypeptide termini. Additionally, some models were not complete, for instance Grimsley et al. [15] did not provide *pKa* value for arginine. Similarly, Dawson model did not include the charge of terminal groups. Therefore, the missing values were introduced (by taking average from other *pKa* values or similar sets) in order to improve the results (the models with less than nine parameters always performed worse than those having at least all nine parameters, see for instance the results for Patrickios, six-parameter model).

**Table 4:** Most commonly used *pKa* values for the ionizable groups of proteins. Note that Bjellqvist and ProMoST use different amounts of additional *pKa* values (not shown), which take into account the relative position of the ionized group (whether it is located on the N- or C- terminus or in the middle). For more details, see References 4 and 5 and the “Theory” section on the IPC web site

Amino acid	NH_2_	COOH	C	D	E	H	K	R	Y
EMBOSS [29]	8.6	3.6	8.5	3.9	4.1	6.5	10.8	12.5	10.1
DTASelect [30]	8	3.1	8.5	4.4	4.4	6.5	10	12	10
Solomons [31]	9.6	2.4	8.3	3.9	4.3	6	10.5	12.5	10.1
Sillero [32]	8.2	3.2	9	4	4.5	6.4	10.4	12	10
Rodwell [33]	8	3.1	8.33	3.68	4.25	6	11.5	11.5	10.07
Patrickios [34]	11.2	4.2	-	4.2	4.2	-	11.2	11.2	-
Wikipedia	8.2	3.65	8.18	3.9	4.07	6.04	10.54	12.48	10.46
Lehninger [35]	9.69	2.34	8.33	3.86	4.25	6	10.5	12.4	10
Grimsley [15]	7.7	3.3	6.8	3.5	4.2	6.6	10.5	12.04^a^	10.3
Toseland [36]	8.71	3.19	6.87	3.6	4.29	6.33	10.45	12	9.61
Thurlkill [37]	8	3.67	8.55	3.67	4.25	6.54	10.4	12	9.84
Nozaki [38]	7.5	3.8	9.5	4	4.4	6.3	10.4	12	9.6
Dawson [39]	8.2^b^	3.2^b^	8.3	3.9	4.3	6	10.5	12	10.1
Bjellqvist [16]	7.5	3.55	9	4.05	4.45	5.98	10	12	10
ProMoST [17]	7.26	3.57	8.28	4.07	4.45	6.08	9.8	12.5	9.84
IPC_protein	9.094	2.869	7.555	3.872	4.412	5.637	9.052	11.84	10.85
IPC_peptide	9.564	2.383	8.297	3.887	4.317	6.018	10.517	12.503	10.071

^a^Arg was not included in the study, and the average *pKa* from all other *pKa* sets was taken

^b^NH2 and COOH were not included in the study, and they were arbitrary taken from Sillero set

### Datasets

The aim of the present study was to derive computationally more accurate *pKa* sets using currently available data. For training and validation, the following datasets were used:

- The IPC peptide *pKa* set was optimized using peptides from three, high-throughput experiments:
  - unmodified 5,758 peptides from Gauci et al. [18] – peptides from zebrafish lysate fractionated using isoelectric focusing
  - PHENYX dataset (7,582 peptides) [4] – peptides from Drosophila Kc167 cell line fractionated using isoelectric focusing on off-gel electrophoresis device
  - SEQUEST dataset (7,629 peptides) [4] – peptides from Drosophila Kc167 cell line fractionated using isoelectric focusing on off-gel electrophoresis device
- The IPC protein *pKa* set was optimized using proteins from two databases:
  - SWISS-2DPAGE, release 19.2 (2,530 proteins) [19] – based on the literature data about *pI* linked to UNIPROT accession numbers
  - PIP-DB (4,947 entries) [20] – based on literature data, provide *pI* and sequence information for about half of the records (for details see Table 5).

**Table 5:** Detailed statistics for the available datasets

Dataset	Initial no. entries	No. entries with sequence and pI	No. entries after removing outliers	No. entries after removing duplicates
Gauci et al.	5,758	5,758	NA	NA
PHENYX	7,582	7,582	NA	NA
SEQUEST	7,629	7,629	NA	NA
IPC_peptide	-	20,969	20,969	16,882 [25] [75]
SWISS-2DPAGE	2,530	1,054	1,029	982
PIP-DB	4,947	2,427	2,254	1,307
IPC_protein	-	3.481	3,283	2,324 [25] [75]

*NA* not available refers to the situation where the given dataset was not created because a merged version was used

Note: all datasets presented in the table are available as hyperlinks; the final datasets were divided randomly into 75 % training and 25 % testing subsets (denoted as [75] and [25], respectively)

First, the raw data from the individual datasets was parsed to the unified fasta format with information about the isoelectric point stored in the headers. Next, datasets consisting of proteins and datasets consisting of peptides were merged into two datasets (IPC_protein and IPC_peptide, respectively). The data was carefully validated, e.g., if multiple experimental *pI* values were reported, the average was used. The first, major splicing form of the protein (most widely expressed) taken from UniProt [21] was used for SWISS-2DPAGE. None information about experimental methods used for obtaining isoelectric points or their specificity was used implicitly during this study. Similarly, as the information about post translational modifications (PTMs) was not included directly in SWISS-2DPAGE and PIP-DB, it was not possible to investigate in detail PTMs contribution to *pI* and they were assumed to be absent. Outliers representing possible annotation errors in databases were removed (proteins with mean standard error (MSE) > 3 between the experimental isoelectric point and the average predicted *pI*; note that under this cutoff, no peptides were removed; it should be stressed that removed outliers do not differ from other proteins with the respect of amino acid content, predicted protein disorder [22] and secondary structure [23], for details see Additional file 1: Table S4). Next, redundant data was removed using CD-HIT [24] (0.99 sequence identity threshold was used; in this case, it was adequate to use such a high sequence identity because even single mutations in the charged residues can lead to dramatic changes in *pI*; moreover other sequence identity thresholds gave similar results; data not shown). This step also removed duplicates (multiple entries assigned to the same sequence coming from two different databases). Finally, 25 % of the randomly chosen proteins and peptides were excluded for final testing, and the remaining 75 % were used for 10-fold cross-validated training.

Detailed statistics for the datasets can be found in Table 5. The main dataset files are available as Additional files 2 and 3 and/or online in the “Datasets” section of the IPC web site.

### Calculation of the isoelectric point

As noted before, the isoelectric point is determined by iteratively calculating the sum of Eqs. 1 and 2 for the individual charged groups for a given pH. The calculation can be performed exhaustively, but this would not be practical. Instead, the bisection algorithm [25] is used, which in each iteration halves the search space (initially, the pH is set to 7) and then moves higher or lower by 3.5 (half of 7) depending on the charge. In the next iteration, the pH is changed by 1.75 (half of 3.5), and so on. This process is repeated until the algorithm reaches the desired precision. Bisection improves the speed by 3–4 orders of magnitude, and after approximately a dozen of iterations, the algorithm converges with 0.001 precision. Next, the speed improvement can be obtained by starting the search from a rough approximation of the solution rather than 7 (in this case, a pH of 6.68 was used, which is the average isoelectric point for approximately 318,000 proteins taken from the *SwissProt* database [26], 90 % sequence identity threshold was used).

### Performance measures

To measure the performance, two metrics were used i.e., the root-mean-square deviation (RMSD) and the number of outliers, defined as *pI* predictions with a mean standard error (MSE) larger than the given threshold in comparison with the experimental *pI.* To remove potential outliers, for the protein datasets, an MSE of three was used, and for peptide datasets, an MSE of 0.25 was used. Moreover, for the preliminary analysis, the Pearson correlation was used.

### Optimization

The optimization procedure was designed to obtain nine optimal *pKa* values (corresponding to the N- and C-termini and the C, D, E, H, K, R, and Y charges). The cost function was defined as the root-mean-square deviation (RMSD) between the true isoelectric points from the available datasets and those calculated using the new *pKa* set(s). Optimization was performed using a basin-hopping procedure [10] which uses a standard Monte Carlo algorithm with Metropolis criterion to decide whether to accept a new solution. The previously published *pKa* values were used as the initial seeds. To limit the search space, a truncated Newton algorithm [27] was used, with 2 pH unit bounds for the *pKa* variables (e.g., if the starting point for Cys *pKa* was 8.5, the solution was allowed in the interval [6.5, 10.5]). The optimization was run iteratively multiple times using intermediate *pKa* sets until the algorithm converged and no better solutions could be found. To avoid overfitting, both the IPC_protein and IPC_peptide datasets were randomly divided into 75 % training datasets (used for *pKa* optimization) and 25 % testing datasets (not used during optimization). During training, nested 10-fold cross-validation was used [28]. Thus, the IPC was optimized separately on k-1 partitions and tested on the remaining partition. The training was repeated ten times in all combinations. The resulting *pKa* sets were averaged. In general, this process resulted in slower convergence of the algorithm and a longer training time but prevented overfitting. Apart from the nine-parameter model (nine *pKa* values for charged residues) also more advanced models similar to Bjellqvist and ProMoST were also tested. Their performance was on a similar level thus the simpler, nine-parameter model was used in the final version of IPC.

### Implementation

**Fig. 4:**
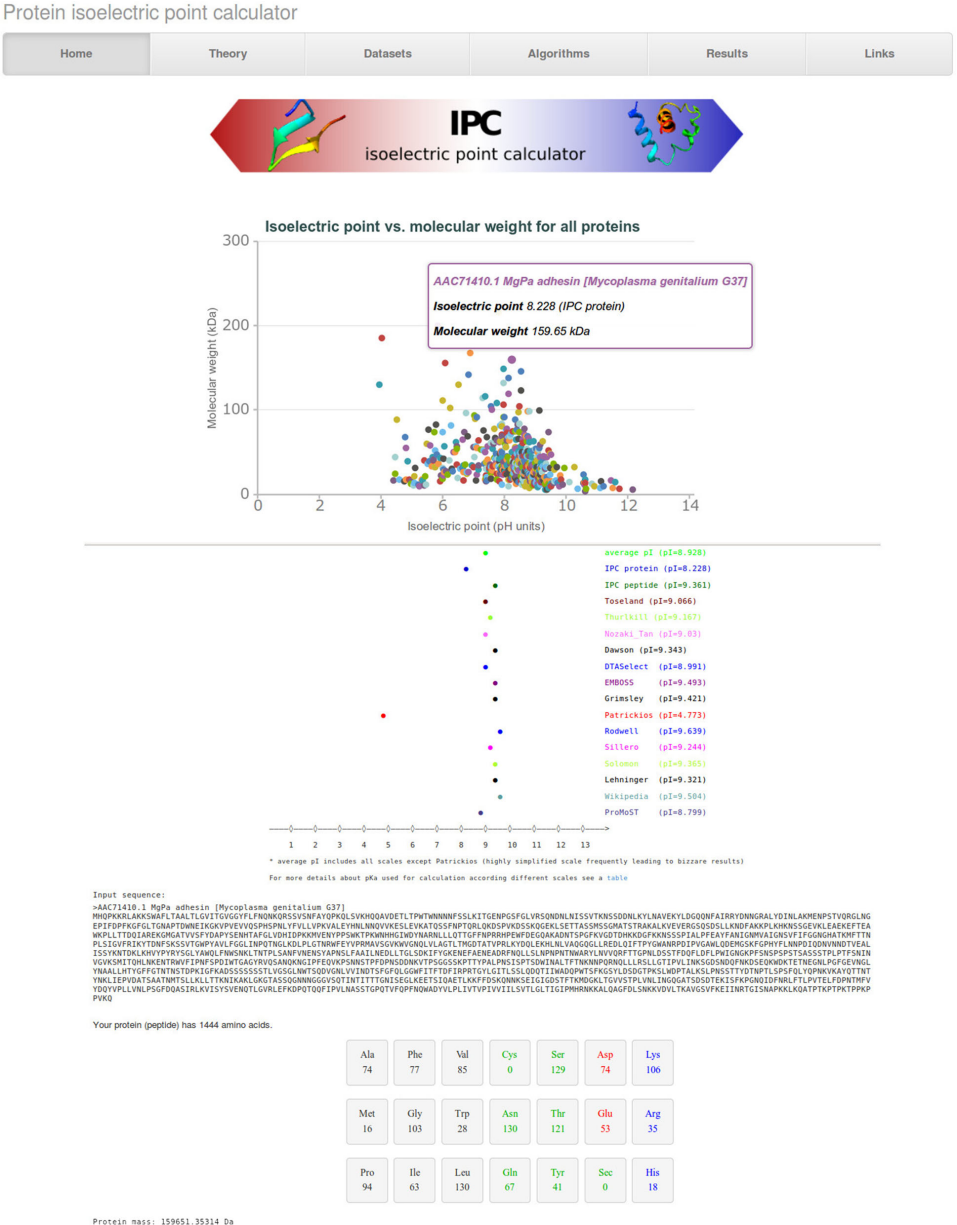
Exemplary output of the IPC calculator for the *Mycoplasma genitalium* G37 proteome (476 proteins). The scatter plot with the predicted isoelectric points versus molecular weight for all proteins is presented at the top. Then, for individual proteins, *pI* predictions based on different *pKa* sets are presented alongside the molecular weight and amino acid composition

The IPC, Isoelectric Point Calculator is available as a web server (Fig. 4) implemented in PHP server-side scripting language. Additionally, HTML5 JavaScript charting library CanvasJS (http://canvasjs.com) and bootstrap (http://getbootstrap.com) were used. Moreover, IPC can be used on any operating system as a standalone program written in Python language (Additional file 4).

## Reviewers’ comments

### Reviewer’s report 1

Frank Eisenhaber, Bioinformatics, A*STAR’s Biomedical Sciences Institute

## Reviewer comments

### Reviewer summary

The author reviews the state of the art in the *pI* computation from protein sequence, provides an improved software tool and presents a WWW site with lots of related information, a WWW server and the software download.

### Reviewer recommendations to authors

This is a very carefully prepared MS that can be published as is.

Minor issues

n/a

Authors’ response: *I thank the reviewer for highlighting the general interest of presented tool and his positive reaction to the manuscript*

### Reviewer’s report 2

Zoltán Gáspári, Pazmany University, Budapest

## Reviewer comments

### Reviewer summary

The manuscript describes a novel set of pKa values for peptides and proteins. The set can be used to estimate the isoelectric point of these macromolecules. The problem is of importance in protein/peptide studies and improvements in the pKa data sets used can be useful.

Authors’ response: *I thank the reviewer for his supportive comments of the study and for highlighting the general interest of presented findings. I have made a concerted effort to address all of his concerns.*

Reviewer recommendations to authors

Major recommendations:

- It is really interesting that the prediction works better for prokaryotic than for eukaryotic proteins. Can the author perform a bit more detailed analysis on this topic besides pointing out the role of PTMs? Do the worst outliers exhibit characteristic amino acid distributions? for example, eukaryotic proteomes are abundant in intrinsically disordered proteins for which the peptide data set might yield better results in some cases.

Authors’ response: *I am most grateful to the Reviewer for bringing this point to my attention. To address Reviewer’s comment I performed additional analysis (included in the supplement as Additional file* *1* *: Table S4). There are 195 outliers (sequences for which pI delta of MSE > 3) vs 2,324 non-outliers (protein sequences which are used for testing and training). Additionally, I have chosen 195 randomly selected sequences from non-outliers, to be sure that sample size does not matter. Conclusions: the outliers are usually shorter and slightly more disordered, but this is not statistically significant. Similarly secondary structure composition and charged amino acid frequencies are very similar. As the result of this analysis does not introduce any new, unexpected information, I added it only to the supplement and mentioned briefly in* *methods* *section, where I defined outliers (lines 311–313). *

- It could be interesting if the author could give any further insights into the variations of the pKa values in the sets and especially the divergence of the newly suggested values relative to those in the literature. There is already a discussion of this in the manuscript just before the Conclusions section but as it is both an important and an interesting aspect, the manuscript might benefit from a more detailed analysis of this question.

Authors’ response: *I decided that longer discussion about this topic would be too technical and too speculative, and after all it would not change the results and I doubt that this will be interesting for broad readership. Additionally, it would not improve the flow of the manuscript (this is rather off topic). Nevertheless, I also think that it is interesting aspect, thus I added this information to Additional file* *1* *: Table S3 underscoring the most divergent values and briefly discussing it possible source*.

A short description of the origin of the data sets used could also be helpful for the reader.

Authors’ response: *The asked information can be found in lines 288–299 in which the Reviewer can read about the organism and technique used for the generation of peptide sets, and references to original studies from which data had been taken. Moreover, all original files for datasets are available as hyperlinks from first column of the Table* *5* *and also from* *http://isoelectric.ovh.org/datasets.html* *– in case if they would be not available in the future from their source urls. For proteins, the information about the experimental technique and the organism is available only partially (see e.g.* *http://isoelectric.ovh.org/datasets/ch2d19_2.dat* *). In any case, those data were not used directly during the datasets construction or optimization not to favor any technique or an organism. For instance, for protein dataset most proteins comes from eukaryotic organisms, 1455 sequences versus 837 sequences coming from Prokaryotes. More detailed data about organism distribution can be seen on the pie plots in the supplement (Additional file* *1* *: Figure S1). In the nutshell, most of the protein sequences come from human, E. coli, S. aureus, R. norvegicus, M. musculus and yeast. Moreover, PIP-DB in this respect is more diverse having data from multiple organisms. Unfortunately, similar analysis for the methods tag is not possible as this tag is not very informative (for SWISS-2DPAGE 2124/2186 entries are tagged as “MAPPING ON GEL” and for PIP-DB 2007/2427 entries are tagged as different versions of isoelectric focusing).*

*I think that current, brief description the Reviewer can find in lines 288–299 is sufficient and more detailed descriptions of the methods from the original studies is out of the scope of presented manuscript and would extend the manuscript unnecessarily with minor benefit for the Readers.*

Minor recommendations:

- The author states that when multiple data were available for the isoelectric point, the average was taken. It would be nice to know how divergent these data were and whether the author has any hints on whether this affects the performance in any detectable way.

Authors’ response: *The information about the divergence is available in the headers of the fasta files e.g.* *http://ipc.netmark.pl/datasets/pip_ch2d19_2_1st_isoform_outliers_3units_cleaned_0.99.fasta* *contains:*

>P04807-1|[′5.17/55102′, ′5.27/54793′]

MVHLGPKKPQARKGSMADVPKELMQQIENFEKIFT....

*This record comes from SWISS-2DPAGE database and the header means that two pI measurements are known: 5.17 and 5.27. Moreover, it can be noticed that reported molecular weights (55.1 and 54.8 kDa) differ from predicted 53.9 kDa which could indicate that this sequence contains post translational modifications which may or may not influence the isoelectric point (neither SWISS-2DPAGE or PIP-DB database contains information about the modifications), but indirectly it can be seen by molecular weight increase, other possible bias may come from the technique used to measuring pI and molecular weight or any random factors between measurements.*

*My attempt to deal with the possible noise in the data was as follows:*

- *include as many measurements as possible preferably coming from different databases*
- *use the average of the measurements*
- *as even after averaging the pI for some of sequences deviates highly from the average predicted pI (Fig.* 1 *) I decided to investigate how much this could be explained by possible annotation errors in the databases. I re-checked randomly selected records with the biggest deviation between experimental and theoretical pI and their source publications until I stop to find obvious annotation errors (in this way I set a threshold on MSE > 3 for removing outliers)*.

*To sum up this part of the comment, the primary databases used for the construction of protein and peptide datasets have different quality. They may contain multiple annotation errors, but the only possible thing I could do in high-throughput and automatic way is to minimize the effect of this noise (see for instance Table* 3 *) by averaging the multiple measurements and removing the obvious errors identified by comparison of experimental and theoretical pI. In other words, it was not possible in reasonable time, to verify correctness of > 2,000 entries (available in already published and widely used databases) referenced in many times by multiple publications one by one. Table* 3 *shows that the datasets used have strong influence on the accuracy of the method (per value), but in most cases the order of the methods stay the same or is very similar which indicates that even in the noisy data the methods are capable to detect signal.*

- In the 10-fold cross-validation process, how divergent were the resulting pKa sets that were averaged? What is the relation of this divergence to the diversity in the other data sets?

Authors’ response: *From the observed divergence I would rather speculate that the landscape of the search space is quite flat with multiple local minima. There are many possible 9 sets of pKa values which produce only slightly worse results. Therefore, the optimization was run 2,000 times to allow for exploring the search space in the different places and the local minimum was refined by bashing-hopping.*

- Please provide a short explanation (in the Methods section) of the asterisked comments for Table 4 and Additional file 1: Table S3 (e.g. why the Sillero terminal pKa values were chosen to complete for the Dawson data set).

Authors’ response: *done as suggested, in both cases adding extra pKa values not included in original studies improved the results. Having the initial results from Patrickios, six-parameter model it was obvious that skipping Arg or terminal charges will have detrimental effect on the performance thus I decided to add them ad hoc, these values were taken as the average from few scales or most similar scale I know at the time of doing that (initially there were only 6–7 scales used, but over the years I implemented more and more scales).*

### Minor issues

- The language of the manuscript needs careful revision. Most of the concepts can be deduced from the present version but the phrasing should be done with more care. So, although I think that the paper can be understood in its present form, I strongly recommend extensive language editing before final publication. Some examples: - “nine parametric model” for me would mean nine distinct models which are all parametric. Maybe the term “nine-parameter model” would be more appropriate (meaning a single model with 9 parameters). - “Basin-Hopping”: as this does not refer to names, simply “basin-hopping” can be written. - page 8, lines 203 and 233: instead of positively and negatively charged macromolecules, the author means residues here?

Authors’ response: *I apologize for the problematic phrasing of some of the sentences. I hope that the corrected version of the manuscript is better.*

- For the additional FASTA files some explanation of the information in the headers would be welcome.

Authors’ response: *As requested I added in all FASTA files more information at the beginning about the content of the headers and how they should be interpreted (available as hyperlinks from three, right columns in the Table* *5* *and also from* *http://isoelectric.ovh.org/datasets.html* *19 files in total). Although, the headers could be simplified and in current version they may have different form depending from which source they come from I decided to leave them as they are (even if sometimes they seems to be hard to understand immediately) as it is easy to check the correctness of the parsing in comparison to original files.*

## Additional files

Additional file 1: Table S1. Performance of isoelectric point prediction algorithms on prokaryotic (837 proteins) and eukaryotic (1,455 proteins) datasets derived from SWISS-2DPAGE and PIP-DB. **Table S2. **Performance of isoelectric point prediction algorithms on proteins having only one splicing isoform. **Table S3.** Statistical comparison of previous pKa values to IPC_protein and IPC_peptide sets. **Table S4.** Statistical comparison of outliers in protein dataset. **Figure S1.** Organism distribution of sequences from SWISS-2DPAGE and PIP-DB. (PDF 409 kb)
Additional file 2: IPC peptide dataset (16,882 peptides, derived from Gauci et al. PHENYX and SEQUEST after 99 % redundancy removal) – fasta formatted. (FASTA 999 kb)
Additional file 3: IPC protein dataset (2,324 proteins, derived from SWISS-2DPAGE and PIP-DB after 99 % redundancy removal) – fasta formatted. (FASTA 969 kb)
Additional file 4: IPC Isoelectric Point Calculator source code (python, any OS). (TXT 39 bytes)

## Abbreviations

2D-PAGE: 2-D polyacrylamide gel electrophoresis
ANN: Artificial neural networks
IPC: Isoelectric point calculator
MS: Mass spectrometry
MSE: Mean standard error
PDB: Protein data bank
*pI*: Isoelectric point
*pKa*: Dissociation constant
PTM: Posttranslational modification(s)
R^2^: Pearson correlation
RMSD: Root-mean-square deviation
SVM: Support vector machines
