# Supplementary material for: IPC – Isoelectric Point Calculator
Source: Biol Direct. 2016 Oct 21;11:55. doi: 10.1186/s13062-016-0159-9 (PMC5075173; doi:10.1186/s13062-016-0159-9)
Supplement: Additional file 1: Table S1. — Performance of isoelectric point prediction algorithms on prokaryotic (837 proteins) and eukaryotic (1,455 proteins) datasets derived from SWISS-2DPAGE and PIP-DB. Table S2. Performance of isoelectric point prediction algorithms on proteins having only one splicing isoform. Table S3. Statistical comparison of previous pKa values to IPC_protein and IPC_peptide sets. Table S4. Statistical comparison of outliers in protein dataset. Figure S1. Organism distribution of sequences from SWISS-2DPAGE and PIP-DB. (PDF 409 kb) [file 13062_2016_159_MOESM1_ESM.pdf]

IPC – Isoelectric Point Calculator, Lukasz P. Kozlowski

Additional file 1

Table S1. Performance of isoelectric point prediction algorithms on prokaryotic (837 proteins) and eukaryotic (1455 proteins) datasets derived from SWISS-2DPAGE and PIP-DB.

	Eukaryote			Prokaryote		
Method	RMSD	%	Outliers	RMSD	%	Outliers
IPC_protein	0.945	0	130	0.629	0	24
Toseland	1.002	14	147	0.707	19.6	33
Bjellqvist	1.048	26.8	164	0.651	5.2	27
ProMoST	1.035	23.1	171	0.682	13.2	37
Dawson	1.038	24	166	0.712	21.1	41
Wikipedia	1.048	26.9	172	0.713	21.4	40
Rodwell	1.035	22.9	163	0.762	35.8	41
Grimsley	1.065	31.7	174	0.702	18.4	39
Solomon	1.065	31.9	174	0.702	18.5	40
Lehninger	1.050	27.4	165	0.709	20.4	22
Nozaki	1.141	57.1	194	0.710	20.6	27
Thurlkill	1.135	55	189	0.769	38.2	40
DTASelect	1.135	55	186	0.772	39.1	35
EMBOSS	1.159	63.8	201	0.792	45.8	52
Sillero	1.175	69.9	211	0.758	34.7	36
Patrickios	2.537	3807.8	688	1.731	1165.1	169
Avg_pI*	1.056	29.1	169	0.706	19.5	35

* Average from all *pKa* sets without the Patrickios (highly simplified *pKa* set) and IPC sets. Note, that the average *pI* is calculated on the level of individual protein or peptide

Table S2. Performance of isoelectric point prediction algorithms on proteins having only one splicing isoform. In total, according Uniprot out of 2,324 proteins from the main dataset only 148 proteins (6.4%) have additional isoforms (45 proteins, 7.7% in the test dataset respectively).

Method	Test and train dataset (2,106 proteins)			Test dataset (536 proteins)		
	RMSD	%	Outliers	RMSD	%	Outliers
IPC_protein	0.848	0.0	150	0.875	0.0	44
Toseland	0.908	14.8	165	0.933	14.3	46
Bjellqvist	0.915	16.8	170	0.941	16.4	44
Dawson	0.917	17.3	187	0.941	16.5	52
Wikipedia	0.928	20.3	191	0.950	19.0	52
Rodwell	0.935	22.3	192	0.959	21.3	55
Grimsley	0.945	25.0	184	0.965	23.2	53
Solomon	0.943	24.5	191	0.966	23.4	54
Lehninger	0.944	24.7	193	0.967	23.6	55
ProMOST	0.936	22.7	169	0.964	22.8	48
Nozaki	0.991	39.0	187	1.016	38.4	51
Thurlkill	1.005	43.8	201	1.023	40.6	54
DTASelect	1.006	44.0	194	1.026	41.8	52
EMBOSS	1.029	51.7	222	1.048	49.0	61
Sillero	1.028	51.4	213	1.051	49.9	57
Patrickios	2.296	2710.9	778	2.391	3183.0	204
Avg_pI*	0.936	22.6	181	0.959	21.4	47

Table S3. Statistical comparison of previous pKa values to IPC_protein and IPC_peptide sets. The differences bigger than one sigma were underlined).

Amino acid	NH2	COOH	C	D	E	H	K	R	Y
EMBOSS	8.6	3.6	8.5	3.9	4.1	6.5	10.8	12.5	10.1
DTASelect	8	3.1	8.5	4.4	4.4	6.5	10	12	10
Solomon	9.6	2.4	8.3	3.9	4.3	6	10.5	12.5	10.1
Sillero	8.2	3.2	9	4	4.5	6.4	10.4	12	10
Rodwell	8	3.1	8.33	3.68	4.25	6	11.5	11.5	10.07
Patrickios	11.2	4.2	-	4.2	4.2	-	11.2	11.2	-
Wikipedia	8.2	3.65	8.18	3.9	4.07	6.04	10.54	12.48	10.46
Lehninger	9.69	2.34	8.33	3.86	4.25	6	10.5	12.4	10
Grimsley	7.7	3.3	6.8	3.5	4.2	6.6	10.5	12.04*	10.3
Toseland	8.71	3.19	6.87	3.6	4.29	6.33	10.45	12	9.61
Thurlkill	8	3.67	8.55	3.67	4.25	6.54	10.4	12	9.84
Nozaki	7.5	3.8	9.5	4	4.4	6.3	10.4	12	9.6
Dawson	8.2**	3.2**	8.3	3.9	4.3	6	10.5	12	10.1
Bjellqvist	7.5	3.55	9	4.05	4.45	5.98	10	12	10
ProMoST	7.26	3.57	8.28	4.07	4.45	6.08	9.8	12.5	9.84
Average	8.42	3.33	8.32	3.91	4.29	6.23	10.5	12.06	10.0
Std. Dev.	1.04	0.49	0.73	0.23	0.13	0.24	0.43	0.37	0.23
**IPC_protein**	**9.094**	**2.869**	**7.555**	**3.872**	**4.412**	**5.637**	**9.052**	**11.84**	**10.85**
Sigma diff.	-0.65	0.93	1.05	0.16	-0.93	2.48	3.36	0.63	-3.66
**IPC_peptide**	**9.564**	**2.383**	**8.297**	**3.887**	**4.317**	**6.018**	**10.517**	**12.503**	**10.071**
Sigma diff.	-1.1	1.92	0.03	0.09	-0.2	0.89	0.0	-1.1	-0.3

*Arg was not included in the study, and the average *pKa* from all other *pKa* sets was taken.
** NH2 and COOH were not included in the study, and they were arbitrary taken from Sillero set.

Table S4. Statistical comparison of outliers in protein dataset. Both SWISS-2DPAGE and PIP-DB were cleaned of outliers (MSE > 3 between experimental *pI* and average predicted *pI;* 195 sequences). Their length, protein disorder and secondary structure content, and charged residues composition were compared to final protein dataset (denoted in the table as "non-outliers"). No statistical significant differences were noticed.

	**Outliers**	**195 random non-outliers**	**Non-outliers**
No. of sequences	195	195	2,324
No. of aa	54,051	73,277	899,524
Length	277 ± 211	375 ± 232	387 ± 243
% of disorder	23.5	22.2	20.6
% of helix	31.3	30.4	29.0
% of sheet	16.8	17.8	17.9
% of coil	51.9	51.8	53.1
% of K	6.74	6.37	6.54
% of E	5.67	5.81	5.93
% of R	5.21	5.78	5.87
% of D	5.02	4.59	4.59
% of Y	3.34	3.26	3.28
% of H	2.06	2.28	2.21
% of C	1.54	1.62	1.42

Protein disorder and secondary structure were predicted using RONN and PSIPRED, respectively.

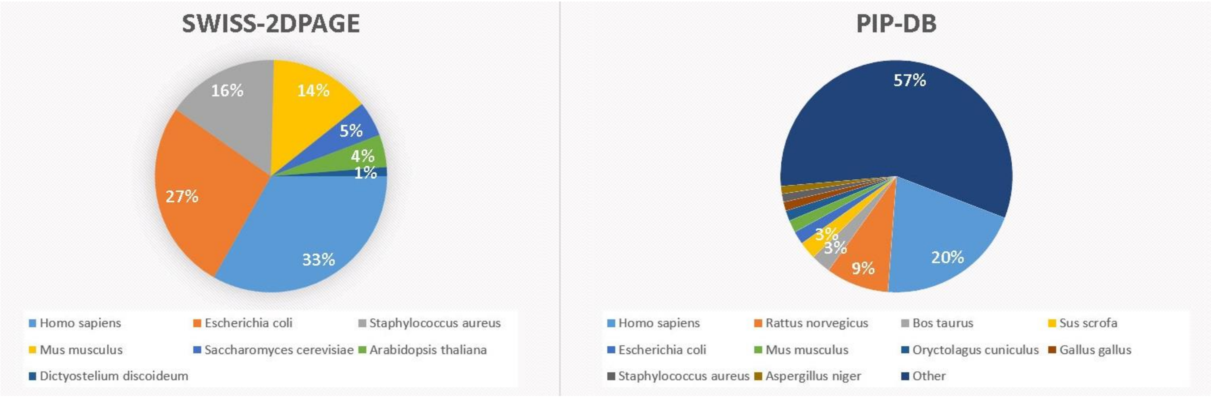

Figure S1. Organism distribution of sequences from SWISS-2DPAGE and PIP-DB. In both databases, the biggest fraction of proteins comes from human, *E. coli, S. aureus, R. norvegicus, M. musculus* and yeast.
